# “I am the coronavirus”: A case report and review

**DOI:** 10.1192/j.eurpsy.2021.788

**Published:** 2021-08-13

**Authors:** J. Camilo, R. Silva, I. Vaz, M. Roque

**Affiliations:** 1 Centro De Gestão De Psiquiatria E Saúde Mental, Centro Hospitalar de Trás-os-Montes e Alto Douro, Vila Real, Portugal; 2 Psiquiatria, Centro Hospitalar de Trás-os-Montes e Alto Douro, Vila Real, Portugal

**Keywords:** COVID-19, coronavirus, psychotic disorder, aripiprazole

## Abstract

**Introduction:**

As disorders of thought, delusions are modified by patients’ background, and so their content varies widely according to location and throughout the ages. The COVID-19 pandemic has shown its global impact on society and mental health of the population, thus becoming a new delusional topic.

**Objectives:**

We report a case where the COVID-19 pandemic has been integrated into a patient’s delusion in an attempt to raise professional awareness for this new psychotic presentation.

**Methods:**

Review of clinical notes and literature review.

**Results:**

A 38-year-old female patient with no prior psychiatric history presented with psychotic symptoms characterized by self-referential ideas, feelings of guilt and delusions of ruin, with a sudden onset of less than 24 hours prior to observation. The patient claimed that she was the coronavirus and, as such, she was a common topic of conversation in both television and social media, and the reported deaths caused by COVID-19 were her own doing. As a result of this, the patient was asking doctors to kill her in order to save everyone else affected by the virus. After evaluation, a diagnosis of Acute and Transient Psychotic Disorder was considered. The patient was initially treated with paliperidone, but due to hyperprolactinemia and menstrual changes this was switched to aripiprazole. Symptoms remitted fully after 21 days of treatment, and six months later no recurrences have been described.

**Conclusions:**

This case illustrates the potential of the coronavirus pandemic outbreak as a new delusional topic. Possible side effects of treatment are also discussed.

